# Aristolochic Acid Induces Renal Fibrosis and Senescence in Mice

**DOI:** 10.3390/ijms222212432

**Published:** 2021-11-18

**Authors:** Shingo Urate, Hiromichi Wakui, Kengo Azushima, Takahiro Yamaji, Toru Suzuki, Eriko Abe, Shohei Tanaka, Shinya Taguchi, Shunichiro Tsukamoto, Sho Kinguchi, Kazushi Uneda, Tomohiko Kanaoka, Yoshitoshi Atobe, Kengo Funakoshi, Akio Yamashita, Kouichi Tamura

**Affiliations:** 1 Department of Medical Science and Cardiorenal Medicine, Yokohama City University Graduate School of Medicine, 3-9 Fukuura, Kanazawa-ku, Yokohama 236-0004, Japan; t186013c@yokohama-cu.ac.jp (S.U.); azushima@yokohama-cu.ac.jp (K.A.); t186042d@yokohama-cu.ac.jp (T.S.); t196004e@yokohama-cu.ac.jp (E.A.); t196042a@yokohama-cu.ac.jp (S.T.); t206042e@yokohama-cu.ac.jp (S.T.); t206044g@yokohama-cu.ac.jp (S.T.); t156025c@yokohama-cu.ac.jp (S.K.); k_uneda@yokohama-cu.ac.jp (K.U.); to_kana8@yokohama-cu.ac.jp (T.K.); tamukou@yokohama-cu.ac.jp (K.T.); 2 Cardiovascular and Metabolic Disorders Program, Duke-NUS Medical School, 8 College Road, Singapore 169857, Singapore; takahiro.yamaji@duke-nus.edu.sg; 3 Department of Neuroanatomy, Yokohama City University School of Medicine, 3-9 Fukuura, Kanazawa-ku, Yokohama 236-0004, Japan; atoyan@yokohama-cu.ac.jp (Y.A.); funako@yokohama-cu.ac.jp (K.F.); 4 Department of Investigative Medicine, Graduate School of Medicine, University of the Ryukyus, 207 Uehara, Okinawa 903-0215, Japan; akyama21@med.u-ryukyu.ac.jp

**Keywords:** chronic kidney disease, renal fibrosis, aristolochic acid, aging, cellular senescence

## Abstract

The kidney is one of the most susceptible organs to age-related impairments. Generally, renal aging is accompanied by renal fibrosis, which is the final common pathway of chronic kidney diseases. Aristolochic acid (AA), a nephrotoxic agent, causes AA nephropathy (AAN), which is characterized by progressive renal fibrosis and functional decline. Although renal fibrosis is associated with renal aging, whether AA induces renal aging remains unclear. The aim of the present study is to investigate the potential use of AAN as a model of renal aging. Here, we examined senescence-related factors in AAN models by chronically administering AA to C57BL/6 mice. Compared with controls, the AA group demonstrated aging kidney phenotypes, such as renal atrophy, renal functional decline, and tubulointerstitial fibrosis. Additionally, AA promoted cellular senescence specifically in the kidneys, and increased renal p16 mRNA expression and senescence-associated β-galactosidase activity. Furthermore, AA-treated mice exhibited proximal tubular mitochondrial abnormalities, as well as reactive oxygen species accumulation. Klotho, an antiaging gene, was also significantly decreased in the kidneys of AA-treated mice. Collectively, the results of the present study indicate that AA alters senescence-related factors, and that renal fibrosis is closely related to renal aging.

## 1. Introduction

With the continuously increasing in lifespan in humans, more individuals are suffering from age-related impairments. Kidneys are typically affected by age-related tissue damage, and the incidence of chronic kidney disease increases with age [1]. Renal aging accelerates overall aging, which results in a shorter lifespan. Therefore, research on renal aging is necessary, although appropriate animal models have not been fully developed. Renal aging is accompanied by various pathological changes, including renal atrophy, glomerulosclerosis, and tubulointerstitial fibrosis [2]. Renal tubulointerstitial fibrosis is the final common pathway in most forms of progressive renal disease, which suggests that renal fibrosis is closely associated with the aged kidney.

In aging research, mice are a reliable tool because of their genetic proximity to humans, the ability to genetically manipulate their genome, and the fact that they present similar aging-related phenotypes to humans during their lifespan [3]. Longitudinal observations using inbred mice are ideal as a model of aging, although it is time-consuming to follow mice for their full lifespan. Additionally, there are several genetically modified mouse models of aging. The Klotho-deficient mouse is a model of premature aging that is used in aging research. However, in this model, renal function is unaffected, and several organs other than the kidneys are impaired [4]. Thus, this mouse model may be inappropriate for research on renal aging.

Aristolochic acid (AA) administration causes a specific type of renal injury known as AA nephropathy (AAN), which is characterized by extensive interstitial fibrosis [5,6]. AA is toxic to the renal tubular epithelium because it promotes the formation of DNA adducts in renal tissues [7]. AA is unlikely to affect organs other than the urinary system and may be useful for the evaluation of kidney-specific alterations. Therefore, AA is commonly used to establish models of renal fibrosis in mice [8,9]. However, it remains unclear whether AA-induced alterations are related to aging in the kidneys. In this study, we examined age-related phenotypes and molecular factors in AAN in mice.

## 2. Results

### 2.1. AA Administration Induced Significant Weight Loss, Renal Atrophy, and a Decline in Renal Function

**Figure 1:**
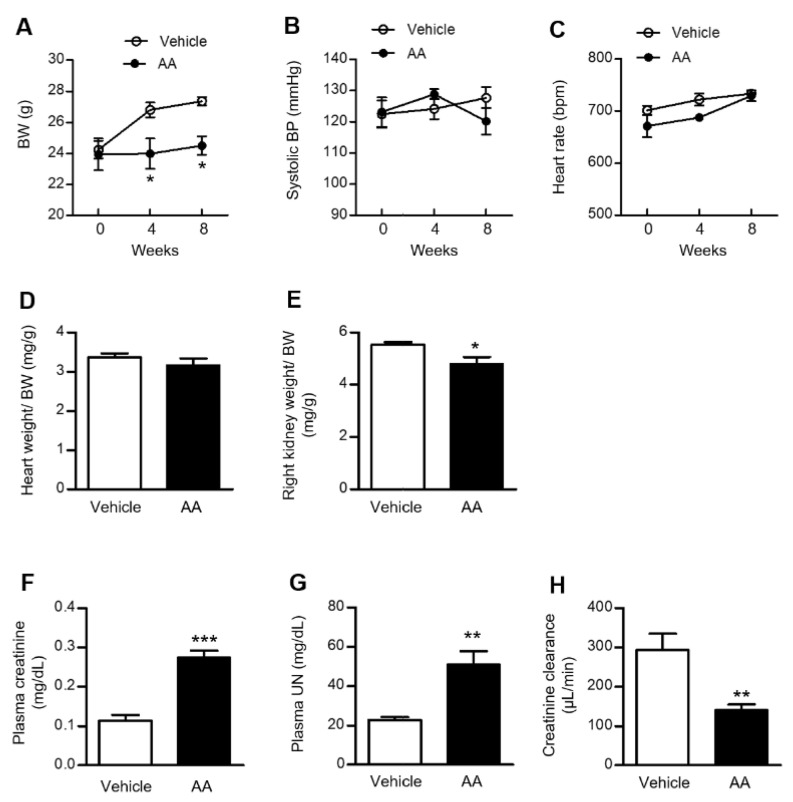
Effects of AA treatment on BW, systolic BP, heart rate, tissue weight, and renal function. (**A**) BW changes in the vehicle-control and AA groups. (**B**) Systolic BP and (**C**) heart rate in the vehicle-control and AA groups at 0, 4, and 8 weeks after the initiation of AA treatment. (**D**) Heart weight/BW and (**E**) kidney weight/BW ratios in the vehicle-control and AA groups at 8 weeks after the initiation of AA treatment. (**F**) Plasma creatinine level, (**G**) plasma UN level, and (**H**) creatinine clearance were measured in the vehicle-control and AA groups at 8 weeks after the initiation of AA treatment. (**A**–**C**) * *p*  <  0.05 compared with the vehicle-control group. Data are presented as mean ± standard error of the mean (SEM) (*n* = 5 per group) and analyzed using two-way ANOVA with Bonferroni’s post-hoc test. (**D**–**H**) * *p*  <  0.05, ** *p*  <  0.01, *** *p*  <  0.001 compared with the vehicle-control group. Data are presented as mean ± SEM (*n* = 4–5 per group) and were analyzed using the unpaired Student’s *t*-test. AA: aristolochic acid; BP: blood pressure; BW: body weight; UN: urea nitrogen.

Baseline body weight (BW) and systolic blood pressure (BP) were identical between the vehicle-control and AA groups. BW in the vehicle-control group increased consistently over 8 weeks. However, weight gain was inhibited by AA administration, and the AA group had significantly lower BW at 4 and 8 weeks after the initiation of AA administration (Figure 1A). There was no significant difference in systolic BP or heart rate between the vehicle-control and AA groups (Figure 1B,C). The heart weight/BW ratio showed no difference between the groups at 8 weeks after the initiation of AA administration (Figure 1D), whereas the kidney weight/BW ratio was significantly lower in the AA group at 8 weeks after the initiation of AA administration (Figure 1E). We next examined renal function in the vehicle-control and AA groups. Plasma creatinine and urea nitrogen (UN) concentrations were significantly higher in the AA group than the vehicle-control group at 8 weeks after the initiation of AA administration. Additionally, AA treatment significantly reduced creatinine clearance compared with the vehicle-control group at 8 weeks after the initiation of AA administration (Figure 1F–H).

### 2.2. AA Administration Induced Overt Tubulointerstitial Fibrosis and Significant Upregulation of Fibrosis-Related Gene Expression in the Kidneys

**Figure 2:**
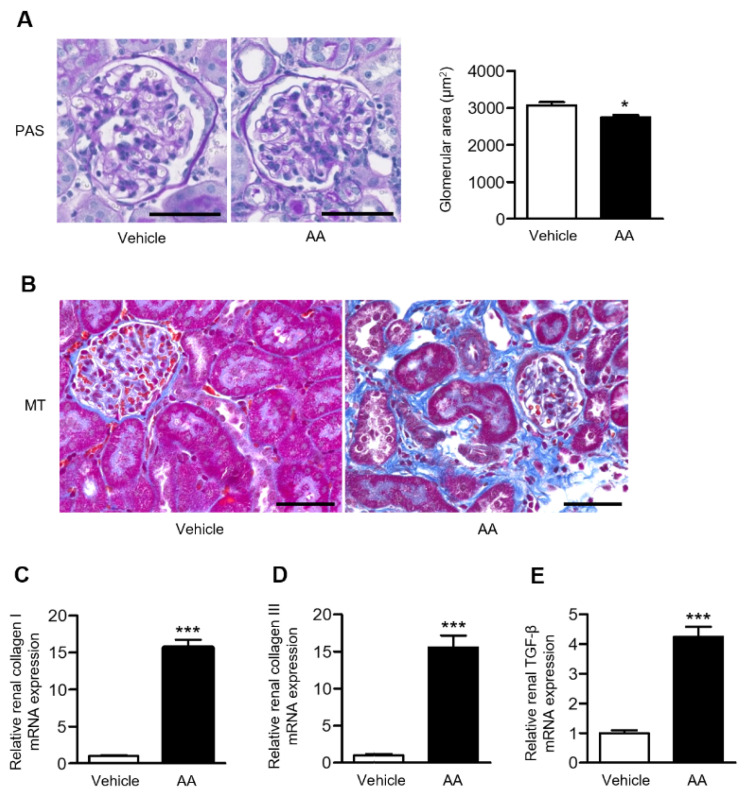
Effects of AA treatment on renal pathological alterations assessed by histological examination and fibrosis-related gene expression. (**A**) Representative images of PAS-stained kidney sections (bar: 50 µm) and the glomerular area in the vehicle-control and AA groups. (**B**) Representative images of MT-stained kidney sections in the vehicle-control and AA groups (bar: 50 µm). Relative renal mRNA expression of (**C**) collagen I, (**D**) collagen III, and (**E**) TGF-β in the vehicle-control and AA groups. (**A**,**C**–**E**) * *p*  <  0.05, *** *p*  <  0.001 compared with the vehicle-control group. Data are presented as mean ± SEM (*n* = 4–5 per group) and were analyzed using the unpaired Student’s *t*-test. AA: aristolochic acid; MT: Masson’s trichrome; PAS: periodic acid-Schiff; TGF-β: transforming growth factor-β.

Histological examination using periodic acid-Schiff (PAS) staining revealed that the glomerular area was significantly reduced in the AA group compared with the vehicle-control group (Figure 2A). Extensive tubulointerstitial fibrosis, assessed using Masson’s trichrome (MT) staining, was observed in the AA group (Figure 2B), as well as with upregulation of the mRNA levels of renal fibrosis-related genes, collagen I and III, and transforming growth factor (TGF)-β (Figure 2C–E). 

### 2.3. AA Administration Accelerated Cellular Senescence, Mitochondrial Dysfunction, and Accumulation of Reactive Oxygen Species (ROS) in the Kidneys

**Figure 3:**
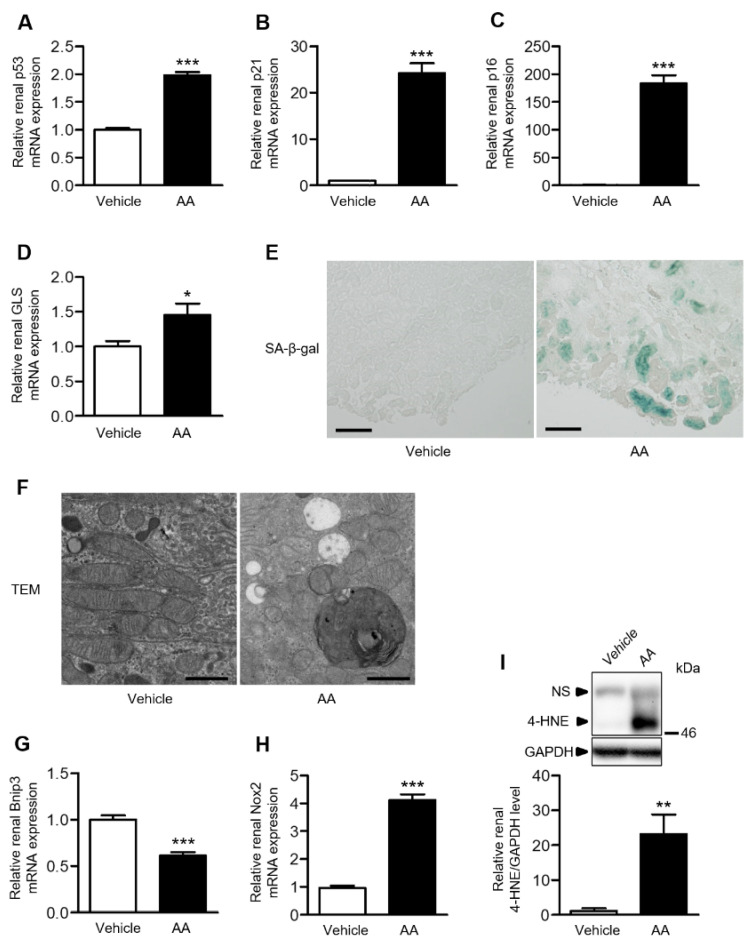
Effects of AA treatment on proximal tubular cells and ROS. Relative renal mRNA expression of (**A**) p53, (**B**) p21, (**C**) p16, and (**D**) GLS. (**E**) Representative images of SA-β-gal-stained kidney sections in the vehicle-control and AA groups (bar: 100 µm). (**F**) Representative TEM images of the proximal tubular cells in the vehicle-control and AA groups (original magnification: ×5000; bar: 1 μm). Relative renal mRNA expression of (**G**) Bnip3 and (**H**) Nox2 in the vehicle-control and AA groups. (**I**) Relative renal 4-HNE level in the vehicle-control and AA groups. (**A**–**D**,**G**–**I**) * *p*  <  0.05, ** *p*  <  0.01, *** *p*  <  0.001 compared with the vehicle-control group. Data are presented as mean ± SEM (*n* = 5 per group) and were analyzed using the unpaired Student’s *t*-test. 4-HNE: 4-hydroxy-2-nonenal; AA: aristolochic acid; Bnip3: BCL2/adenovirus E1B 19-kDa interacting protein 3; GAPDH: glyceraldehyde-3-phosphate dehydrogenase; GLS: glutaminase; NS: nonspecific band; SA-β-gal: senescence-associated β-galactosidase; TEM: transmission electron microscope.

To evaluate cellular senescence, we examined renal mRNA expression of p53, p21, p16, and glutaminase (GLS). The AA group demonstrated significantly higher mRNA levels of these senescence-related genes in the kidneys (Figure 3A–D). Furthermore, the senescence-associated β-galactosidase (SA-β-gal) staining intensity in the kidneys was increased in the AA group and almost absent in the vehicle-control group (Figure 3E). In the AA group, electron microscopy revealed the disappearance of mitochondrial cristae, mitochondrial fragmentation, cytoplasmic vacuolization, and autolysosomes in the proximal tubular cells, concomitant with the downregulation of BCL2/adenovirus E1B 19-kDa interacting protein 3 (Bnip3), a mitochondria-related gene (Figure 3F,G). Renal mRNA expression of Nox2, a component of nicotinamide adenine dinucleotide phosphate (NADPH) oxidase, was significantly increased in the AA group compared with the vehicle-control group (Figure 3H). Furthermore, western blot analysis revealed that the renal 4-hydroxy-2-nonenal (4-HNE) level was significantly increased in the AA group (Figure 3I). These results indicated that AA administration induced cellular senescence, mitochondrial dysfunction, and ROS accumulation in the kidneys.

### 2.4. AA Reduced Renal Klotho Protein Expression

**Figure 4:**
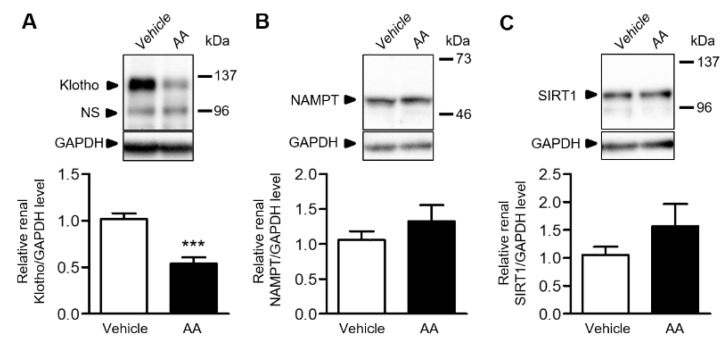
Effects of AA treatment on the renal expression of antiaging proteins. (**A**–**C**) Relative renal protein expression of Klotho, NAMPT, and SIRT1 in the vehicle-control and AA groups. *** *p*  <  0.001 compared with the vehicle-control group. Data are presented as mean ± SEM (*n* = 5 per group) and were analyzed using the unpaired Student’s *t*-test. AA: aristolochic acid; GAPDH: glyceraldehyde-3-phosphate dehydrogenase, NAMPT: nicotinamide phosphoribosyltransferase; NS: nonspecific band; SIRT1: sirtuin1.

We examined the renal expression of antiaging proteins in the vehicle-control and AA groups. Klotho expression was significantly reduced in the AA group compared with the vehicle-control group (Figure 4A), whereas the renal expressions of nicotinamide phosphoribosyltransferase (NAMPT) and sirtuin1 (SIRT1) was similar between the groups (Figure 4B,C).

## 3. Discussion

To our knowledge, the present study is the first to focus on the evaluation of renal aging-associated alterations in AAN using markers of senescence, such as p16 and SA-β-gal activity. AA treatment promoted renal atrophy, tubulointerstitial fibrosis, and renal functional decline, which were accompanied by upregulation of renal p16 mRNA and SA-β-gal-positive staining. Furthermore, the AA group demonstrated features of renal aging-related mechanisms such as mitochondrial abnormalities, increased oxidative stress, and downregulation of the antiaging gene, Klotho. Taken together, our observations suggest that chronic AA administration partially mimics renal aging.

Cellular senescence is permanent arrest of cell proliferation in response to various stressors, such as DNA damage, and contributes to aging and aging-related diseases. The expression of p16, a cyclin-dependent kinase inhibitor, is correlated with aging in various organs, including the kidneys. Caloric restriction increases lifespan by inhibiting p16 expression [10]. Additionally, elimination of p16-expressing senescent cells in INK-ATTAC mice via injection of AP20187, an FK506-binding protein dimerizer [11], causes attenuation of renal aging phenotypes, such as glomerular sclerosis and renal functional decline. Therefore, p16 is one of the most important aging-related factors. Senescent cells can be detected using SA-β-gal staining, which demonstrates increased β-galactosidase activity at pH 6.0 [12], and is a widely used biomarker of senescent and aged cells. Renal mRNA expression of p16 increases in aged rat kidneys and is accompanied by increased SA-β-gal activity in renal epithelium [13]. In the present study, we demonstrated increased renal p16 mRNA expression and SA-β-gal-staining, indicating that AA induces renal senescence. GLS was recently reported to be essential for the survival of senescent cells via enhanced glutaminolysis and intracellular pH neutralization [14]. Inhibition of GLS-dependent glutaminolysis in aged mice was shown to eliminate senescent cells and ameliorate age-related organ dysfunction. In the present study, upregulation of renal GLS mRNA indicated renal accumulation of senescent cells in the AA group. Thus, chronic AA administration caused cellular senescence in the kidneys.

In addition to cellular senescence, mitochondrial dysfunction is an essential mechanism underlying age-related tissue damage and is accompanied by ROS accumulation [15,16]. Mitochondrial dysfunction drives and maintains cellular senescence [17], whereas cellular senescence contributes directly to mitochondrial dysfunction [18]. Bnip3, located primarily in the outer mitochondrial membrane, may play a role in the regulation of mitophagy in cultured renal proximal tubular cells in response to oxidative stress and hypoxia. In aged mice, caloric restriction increases autophagy in tubular cells, via upregulation of Bnip3, and improves age-induced degeneration of renal function [19]. In the present study, electron microscopy revealed the features of mitochondrial abnormalities that may be affected by reduced renal Bnip3 expression or cellular senescence. Mitochondria are the main intracellular source of ROS. To assess the effects of mitochondrial abnormalities on renal ROS, we examined the renal accumulation of 4-HNE and demonstrated that AA induced ROS accumulation in the kidneys. NADPH oxidases are a major source of ROS. During the acute phase of AAN in mice, renal mRNA expression of Nox2 was elevated, and nitric oxide availability was reduced, which led to sustained hypoxia and ischemia [20]. In aged rat kidneys, accumulation of ROS is accompanied by increased expression of Nox2 [21]. These findings suggest that AA may induce age-related phenotypes via ROS accumulation caused by mitochondrial dysfunctional and upregulation of NADPH oxidases.

Renal Klotho gene expression was decreased in the AA group, whereas the expression of NAMPT and SIRT1 was similar between the groups. Klotho is an antiaging gene that encodes a single-pass transmembrane protein that acts as an aging suppressor. The aging process in Klotho-deficient mice resembled that in humans, including a shorter lifespan, infertility, arteriosclerosis, skin atrophy, osteoporosis, and emphysema [22]. Hypomorphic mutant Klotho mice also exhibited a phenotype of accelerated aging, which was restored by p16 ablation [23]. Since Klotho is an important factor in aging, Klotho gene-modified mice have been widely used in research on aging. However, Klotho gene-modified mice may be inappropriate for research on renal aging owing to the systemic aging-associated impairments of these mice and the fact that renal function is unaffected (i.e., creatinine levels). In contrast, the AAN mouse model may be used as a drug-induced model of renal aging for research purposes, as it has several advantages, such as the relative ease of implementation and the ability to estimate the effects of interventions on renal functional decline.

The present study had some limitations. We evaluated renal aging, focusing mainly on cellular senescence, mitochondrial morphology, and aging-related gene expression. However, the mechanisms of aging are complex and remain poorly understood. Additionally, although Klotho gene expression was decreased in response to AA, we could not demonstrate a causal relationship with the pathological changes in AAN. Further studies are required to determine the roles of the various molecules involved in the development of AAN. Nevertheless, the present study provides novel insights into the use of AAN as a model of renal aging. It is important to note that phenotypic changes in the kidney are strongly associated with lifespan. Although the kidney is easily affected by aging-associated changes, inhibition of renal aging can lengthen overall lifespan. Therefore, further research on renal aging using models of AAN may lead to increased longevity in the future.

## 4. Materials and Methods

### 4.1. Animals

This study was conducted in accordance with the National Institutes of Health guidelines for the use of experimental animals. All animal experiments were approved by the Animal Studies Committee of Yokohama City University (approval Number: FA20-027) and were conducted in compliance with ARRIVE guidelines. Efforts were made to minimize the number of animals used and their suffering. Mice were housed in a controlled environment under a 12-h light/12-h dark cycle at a temperature of 25 °C. Mice had free access to food and water. 

Eight-week-old male C57BL/6 mice were purchased from the Charles River Laboratories (Wilmington, MA, USA) and assigned to the vehicle-control or AA groups following 1 week of acclimatization. Mice were intraperitoneally administered with vehicle or AA (Sigma-Aldrich, St. Louis, MO, USA), which was dissolved in a small amount of dimethyl sulfoxide. Prior to the present study, we conducted a preliminary study using several protocols. The first protocol included administration of AA (3 mg/kg) to C57BL/6 mice intraperitoneally twice a week for 4 weeks without remodeling time; in this protocol, AA caused overt acute renal lesions, such as dilated proximal tubules with loss of brush border (Supplementary Figure S1). In the second protocol, C57BL/6 mice were administered with AA (2.5 mg/kg) intraperitoneally once a week for 4 weeks followed by remodeling time for 4 weeks; plasma creatinine in these mice was 0.19 ± 0.080 mg/dL versus 0.18 ± 0.010 mg/dL (vehicle-control versus AA, respectively; mean ± standard error of the mean (SEM), *p* = 0.86, unpaired Student’s *t*-test, *n* = 4 per group) and plasma UN was 31.3 ± 5.95 mg/dL versus 30.4 ± 3.87 mg/dL (vehicle-control versus AA, respectively; mean ± SEM, *p* = 0.90, unpaired Student’s *t*-test, *n* = 4 per group). Therefore, we determined that this protocol was not suitable for a renal injury model because AA did not induce significant renal functional decline. In the third protocol, C57BL/6 mice were administered with AA (3 mg/kg) intraperitoneally twice a week for 10 weeks without remodeling time; in these mice, the mortality rate in the AA group was 83% (*n* = 6), whereas none of the mice in the vehicle-control group died (*n* = 4). In this protocol, we could not statistically evaluate the renal phenotype induced by AA because of the high mortality. In the fourth protocol, C57BL/6 mice were administered with AA (3 mg/kg) intraperitoneally twice a week for 4 weeks followed by remodeling time for 4 weeks; chronic renal lesions, such as tubulointerstitial fibrosis, and renal functional decline were observed in these mice [9]. Therefore, based on the results of these preliminary investigations, we adopted the fourth protocol to carry out the experiments in the present study.

### 4.2. BP Measurement

Systolic BP and heart rate were measured using the previously described tail-cuff method (BP-Monitor MK-2000; Muromachi Kikai Co., Tokyo, Japan) [24,25]. All measurements were performed between 9:00 and 14:00. At least 10 measurements were performed in each mouse, and the mean value was used for analysis.

### 4.3. Real-Time Quantitative Reverse-Transcription PCR Analysis

Total RNA was extracted from renal tissues using ISOGEN (Nippon Gene, Tokyo, Japan), and complementary DNA (cDNA) was synthesized using the SuperScript III First-Strand System (Invitrogen, Waltham, MA, USA). Real-time quantitative reverse-transcription PCR analysis was performed using the CFX96 Touch Real-Time PCR Detection System (Bio-Rad Laboratories, Hercules, CA, USA), and the reverse transcription products were incubated with TaqMan PCR Master Mix and a custom TaqMan probe (Applied Biosystems, Waltham, MA, USA), as previously described [26]. TaqMan probes against the following genes were used: collagen I (Mm00801666_g1), collagen III (Mm01254476_m1), TGF-β (Mm01178819_m1), p53 (Mm00441964_g1), p21 (Mm04205640_g1), p16 (Mm00494449_m1), GLS (Mm01257297_m1), Bnip3 (Mm01275600_g1), and Nox2 (Mm00627011_m1). mRNA levels were normalized to those of 18S ribosomal RNA.

### 4.4. Western Blot Analysis

Protein expression was analyzed by western blot analysis of the tissue homogenates using a previously described method [27,28]. Total protein extracts were prepared from the tissues using sodium dodecyl sulfate-containing sample buffer. The protein concentration of each sample was measured using the Detergent-Compatible Protein Assay Kit (Bio-Rad Laboratories, Hercules, CA, USA) and the NanoDrop One (Thermo Fisher Scientific, Waltham, MA, USA), with bovine serum albumin as the standard. Equal amounts of protein extracts from the tissue samples were fractionated on 5–20% polyacrylamide gels (ATTO Corp., Tokyo, Japan). Separated proteins were then transferred to polyvinylidene difluoride membranes using the Semi-Dry Transfer System (ATTO Corp., Tokyo, Japan). Membranes were blocked for 1 h at room temperature with phosphate-buffered saline containing 5% skim-milk powder. Membranes were incubated with primary antibodies against Klotho (ab181373 1:1000; Abcam, Cambridge, UK), NAMPT (sc-67020 1:5000; Abcam), SIRT1 (07-131 1:1000, MilliporeSigma, Burlington, MA, USA), 4-HNE (MHN-100P 1:1000; JaICA, Fukuroi, Japan), and glyceraldehyde-3-phosphate dehydrogenase (GAPDH) (2118, 1:2000; Cell Signaling Technology, Danvers, MA, USA). Membranes were washed and incubated with secondary antibodies for 60 min at room temperature. The sites for antibody-antigen reactions were visualized using enhanced chemiluminescence substrate (Merck, Kenilworth, NJ, USA). GAPDH was used as the loading control. Images were analyzed quantitatively using the ChemiDoc Touch (Bio-Rad Laboratories, Hercules, CA, USA).

### 4.5. Histological Analysis

Histological analysis was performed as previously described [29]. Renal tissues from mice were fixed with 4% paraformaldehyde and subsequently embedded in paraffin. Sections (4 µm thick) were stained with PAS and MT stains. To evaluate the glomerular area, 50 glomeruli per mouse were measured and averaged. All images were acquired using the BZ-9000 microscope (Keyence Corp., Osaka, Japan).

### 4.6. SA-β-gal Staining

Renal tissues from mice were rapidly frozen and mounted in Optimal Cutting Temperature compound (Sakura Finetek Japan, Co., Ltd., Tokyo, Japan). Sections (4 μm thick) were prepared using a cryostat (HM550-VPD; Thermo Fisher Scientific) and mounted onto glass slides. SA-β-gal activity was measured using a senescence detection kit (BioVision Inc., Milpitas, CA, USA) according to the manufacturer’s protocols. Samples were viewed under bright field at ×100 magnification using the BZ-9000 microscope (Keyence Corp.).

### 4.7. Electron Microscopy

Electron microscopy analysis was performed as previously described [30]. The mice were anesthetized with isoflurane and perfused through the right aortic arch with heparinized (5 U/mL) physiological saline and 2.5% glutaraldehyde in 0.1 mol/L phosphate buffer at pH 7.4. Specimens for transmission electron microscopy were immersed in 1% osmium tetroxide for 2 h, serially dehydrated in ethanol, and embedded in an Epon mixture. Ultrathin sections were stained with uranyl acetate and lead citrate and examined using the Hitachi H-7500 transmission electron microscope operated at 80 kV (Hitachi, Ltd., Tokyo, Japan). Sections were observed at ×5000 magnification and photographed using a charge-coupled device camera.

### 4.8. Biochemical Analysis

Blood samples were collected by cardiac puncture in the fed state. Whole blood samples were centrifuged at 3000 rpm (MR-150; Tomy Seiko Co., Ltd., Tokyo, Japan) for 10 min at 4 °C to separate the plasma. The resultant plasma samples were stored at −80 °C. Plasma creatinine, UN, and urinary creatinine levels were measured using the Hitachi 7180 autoanalyzer (Hitachi, Ltd., Tokyo, Japan).

### 4.9. Statistical Analysis

Statistical analyses were performed using the GraphPad Prism software (GraphPad Software Inc., San Diego, CA, USA). Data are presented as mean ± SEM. The unpaired Student’s *t*-test was used to compare the differences between the vehicle-control and AA groups. *p* < 0.05 was considered statistically significant.

## 5. Conclusions

AA administration causes kidney senescence, along with tubulointerstitial fibrosis and renal atrophy. The results suggest that renal fibrosis is closely related to renal aging, and that AAN may be useful as a kidney-specific aging model.

## Data Availability

The data presented in this study are available on request from the corresponding author.

## Supplementary Materials

The following are available online at https://www.mdpi.com/article/10.3390/ijms222212432/s1.

## References

[1] Ebert N., Jakob O., Gaedeke J., van der Giet M., Kuhlmann M.K., Martus P., Mielke N., Schuchardt M., Tölle M., Wenning V. (2016). Prevalence of reduced kidney function and albuminuria in older adults: The Berlin Initiative Study. Nephrol. Dial. Transplant..

[2] O’Sullivan E.D., Hughes J., Ferenbach D. (2017). Renal Aging: Causes and Consequences. J. Am. Soc. Nephrol..

[3] Vanhooren V., Libert C. (2013). The mouse as a model organism in aging research: Usefulness, pitfalls and possibilities. Ageing Res. Rev..

[4] Kuro-O M., Matsumura Y., Aizawa H., Kawaguchi H., Suga T., Utsugi T., Ohyama Y., Kurabayashi M., Kaname T., Kume E. (1997). Mutation of the mouse klotho gene leads to a syndrome resembling ageing. Nature.

[5] Jadot I.I., Declèves A.-E., Nortier J., Caron N. (2017). An Integrated View of Aristolochic Acid Nephropathy: Update of the Literature. Int. J. Mol. Sci..

[6] Luciano R.L., Perazella M.A. (2014). Aristolochic Acid Nephropathy: Epidemiology, Clinical Presentation, and Treatment. Drug Saf..

[7] Schmeiser H., Schoepe K.-B., Wiessler M. (1988). DNA adduct formation of aristolochic acid I and II in vitro and in vivo. Carcinogenesis.

[8] Huang L., Scarpellini A., Funck M., Verderio E.A., Johnson T.S. (2013). Development of a Chronic Kidney Disease Model in C57BL/6 Mice with Relevance to Human Pathology. Nephron Extra.

[9] Tsukamoto S., Wakui H., Azushima K., Yamaji T., Urate S., Suzuki T., Abe E., Tanaka S., Taguchi S., Yamada T. (2021). Tissue-specific expression of the SARS-CoV-2 receptor, angiotensin-converting enzyme 2, in mouse models of chronic kidney disease. Sci. Rep..

[10] Krishnamurthy J., Torrice C., Ramsey M.R., Kovalev G.I., Al-Regaiey K., Su L., Sharpless N.E. (2004). Ink4a/Arf expression is a biomarker of aging. J. Clin. Investig..

[11] Baker D.J., Childs B.G., Durik M., Wijers M.E., Sieben C.J., Zhong J., Saltness R.A., Jeganathan K.B., Verzosa G.C., Pezeshki A. (2016). Naturally occurring p16Ink4a-positive cells shorten healthy lifespan. Nature.

[12] Dimri G.P., Lee X., Basile G., Acosta M., Scott G., Roskelley C., Medrano E.E., Linskens M., Rubelj I., Pereira-Smith O. (1995). A biomarker that identifies senescent human cells in culture and in aging skin in vivo. Proc. Natl. Acad. Sci. USA.

[13] Melk A., Kittikowit W., Sandhu I., Halloran K.M., Grimm P., Schmidt B.M., Halloran P. (2003). Cell senescence in rat kidneys in vivo increases with growth and age despite lack of telomere shortening. Kidney Int..

[14] Johmura Y., Yamanaka T., Omori S., Wang T.-W., Sugiura Y., Matsumoto M., Suzuki N., Kumamoto S., Yamaguchi K., Hatakeyama S. (2021). Senolysis by glutaminolysis inhibition ameliorates various age-associated disorders. Science.

[15] Sun N., Youle R.J., Finkel T. (2016). The Mitochondrial Basis of Aging. Mol. Cell.

[16] Perez-Campo R., López-Torres M., Cadenas S., Rojas C., Barja G. (1998). The rate of free radical production as a determinant of the rate of aging: Evidence from the comparative approach. J. Comp. Physiol. B.

[17] Correia-Melo C., Marques F.D.M., Anderson R., Hewitt G., Hewitt R., Cole J., Carroll B.M., Miwa S., Birch J., Merz A. (2016). Mitochondria are required for pro-ageing features of the senescent phenotype. EMBO J..

[18] Passos J.F., Nelson G., Wang C., Richter T., Simillion C., Proctor C.J., Miwa S., Olijslagers S., Hallinan J., Wipat A. (2010). Feedback between p21 and reactive oxygen production is necessary for cell senescence. Mol. Syst. Biol..

[19] Kume S., Uzu T., Horiike K., Chin-Kanasaki M., Isshiki K., Araki S.-I., Sugimoto T., Haneda M., Kashiwagi A., Koya D. (2010). Calorie restriction enhances cell adaptation to hypoxia through Sirt1-dependent mitochondrial autophagy in mouse aged kidney. J. Clin. Investig..

[20] Declèves A.-E., Jadot I., Colombaro V., Martin B., Voisin V., Nortier J., Caron N., Habsch I., De Prez E. (2015). Protective effect of nitric oxide in aristolochic acid-induced toxic acute kidney injury: An old friend with new assets. Exp. Physiol..

[21] Zuo Z., Lei H., Wang X., Wang Y., Sonntag W., Sun Z. (2010). Aging-related kidney damage is associated with a decrease in klotho expression and an increase in superoxide production. AGE.

[22] Kuro-O M. (2008). Klotho as a regulator of oxidative stress and senescence. Biol. Chem..

[23] Sato S., Kawamata Y., Takahashi A., Imai Y., Hanyu A., Okuma A., Takasugi M., Yamakoshi K., Sorimachi H., Kanda H. (2015). Ablation of the p16INK4a tumour suppressor reverses ageing phenotypes of klotho mice. Nat. Commun..

[24] Shigenaga A.-I., Tamura K., Wakui H., Masuda S.-I., Azuma K., Tsurumi-Ikeya Y., Ozawa M., Mogi M., Matsuda M., Uchino K. (2008). Effect of Olmesartan on Tissue Expression Balance Between Angiotensin II Receptor and Its Inhibitory Binding Molecule. Hypertension.

[25] Azushima K., Uneda K., Wakui H., Ohki K., Haruhara K., Kobayashi R., Haku S., Kinguchi S., Yamaji T., Minegishi S. (2019). Effects of rikkunshito on renal fibrosis and inflammation in angiotensin II-infused mice. Sci. Rep..

[26] Wakui H., Tamura K., Masuda S.-I., Tsurumi-Ikeya Y., Fujita M., Maeda A., Ohsawa M., Azushima K., Uneda K., Matsuda M. (2013). Enhanced Angiotensin Receptor-Associated Protein in Renal Tubule Suppresses Angiotensin-Dependent Hypertension. Hypertension.

[27] Ohki K., Wakui H., Kishio N., Azushima K., Uneda K., Haku S., Kobayashi R., Haruhara K., Kinguchi S., Yamaji T. (2018). Angiotensin II Type 1 Receptor-associated Protein Inhibits Angiotensin II-induced Insulin Resistance with Suppression of Oxidative Stress in Skeletal Muscle Tissue. Sci. Rep..

[28] Azushima K., Ohki K., Wakui H., Uneda K., Haku S., Kobayashi R., Haruhara K., Kinguchi S., Matsuda M., Maeda A. (2017). Adipocyte-Specific Enhancement of Angiotensin II Type 1 Receptor-Associated Protein Ameliorates Diet-Induced Visceral Obesity and Insulin Resistance. J. Am. Heart Assoc..

[29] Ohsawa M., Tamura K., Wakui H., Maeda A., Dejima T., Kanaoka T., Azushima K., Uneda K., Tsurumi-Ikeya Y., Kobayashi R. (2014). Deletion of the angiotensin II type 1 receptor–associated protein enhances renal sodium reabsorption and exacerbates angiotensin II–mediated hypertension. Kidney Int..

[30] Takeda A., Atobe Y., Kadota T., Goris R., Funakoshi K. (2015). Axonal regeneration through the fibrous scar in lesioned goldfish spinal cord. Neuroscience.

