# Supplementary material for: Aristolochic Acid Induces Renal Fibrosis and Senescence in Mice
Source: Int J Mol Sci. 2021 Nov 18;22(22):12432. doi: 10.3390/ijms222212432 (PMC8618437; doi:10.3390/ijms222212432)
Supplement: Supplementary file 1 [file ijms-22-12432-s001.zip › ijms-1468524-SI.pdf]

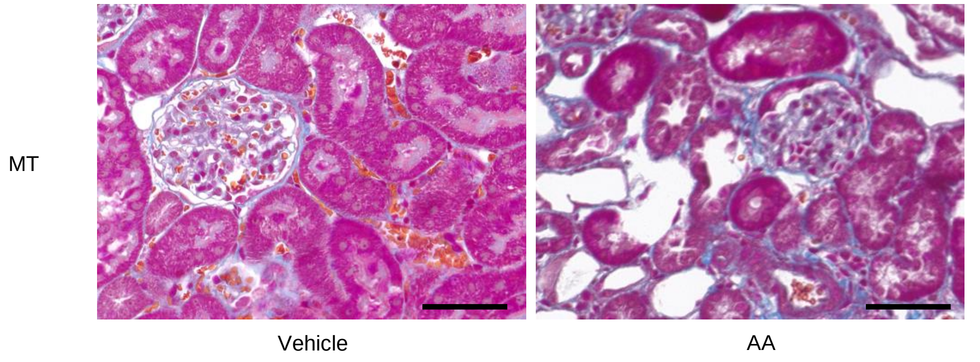

Figure S1. Histological examination of mice administered with AA (3mg/kg) intraperitoneally twice a week for 4 weeks without remodeling time. Representative images of MT-stained kidney sections in the vehicle-control and AA groups (bar: 50 μm). AA: aristolochic acid; MT: Masson's trichrome.
